# A retrospective study of long term follow-up of 2283 vitiligo patients treated by autologous, non-cultured melanocyte–keratinocyte transplantation

**DOI:** 10.18632/aging.202472

**Published:** 2021-02-11

**Authors:** Dimin Zhang, Xiaodong Wei, Weisong Hong, Lifang Fu, Guopei Qian, Ai-e Xu

**Affiliations:** 1 The Department of Dermatology, The Third People’s Hospital of Hangzhou, Hangzhou Institute of Dermatology and Venereology, Hangzhou, China

**Keywords:** vitiligo, non-cultured melanocyte-keratinocyte transplantation

## Abstract

Background: Autologous non-cultured melanocyte-keratinocyte transplantation (MKTP) can be used to treat stable vitiligo cases, but there were insufficient clinical data to evaluate its safety and efficacy.

Objective: To assess the influence of various factors on the therapeutic outcome of MKTP.

Method: The single-center retrospective study included stable vitiligo patients who underwent MKTP between June 2009 and June 2018. Univariate and/or multivariable analysis were used to determine the factors affecting the outcome of repigmentation.

Result: The study comprised 2283 patients who had long-term follow-up data (12-108months). Excellent repigmentation was achieved in 400/606 (66%),788/1341 (58.8%),437/684 (63.9%),18/24 (75%) patients with segmental vitiligo, pre-MKTP phototherapy, younger than 24 years, the lesion on the perineum and scrotum, respectively. However, the patients with a positive family history, Koebner phenomenon responded worse(χ^2^=29.417, P<0.001; χ^2^=107.397, P<0.001; respectively). Overall, a significant positive correlation between duration of stability and percentage of repigmentation was found (χ^2^=42.053, P<0. 001).

Conclusion: MKTP is efficient and well tolerated for stable vitiligo treatment. Various factors such as duration of disease stability, vitiligo type, family history, site of lesion should be carefully assessed before using MKTP, as it would further improve the post-operative repigmentation.

## INTRODUCTION

Vitiligo is an autoimmune skin disease that targets melanocytes and causes patches of depigmentation visible as white spots [1]. Like other autoimmune diseases, vitiligo has extremely complex pathogenesis, including genetic, environmental, and stochastic factors [2]. Vitiligo is the most common skin depigmenting disorder, and it affects approximately 0.5-1% of the world population [3–5]. Vitiligo not only is a devastating disease that affects one’s appearance, but also impacts life quality of the patients [6, 7]. The visible cosmetic defect often leads to psychological problems such as depression and anxiety [7], while causing low self-esteem and social isolation [8, 9]. Among these visible skin sites, the hands and faces are usually affected as well [10], and patients are particularly worried about the spread and deteriorating of the disease at these locations [11].

Many patients seek treatment urgently [10]. However, due to the complex pathogenesis, the management of vitiligo remains challenging [12]. Guidelines have recommended the use of combined medical therapies that include at least one type of light therapy, mainly narrowband (NB) UVB, for the treatment of vitiligo [10]. However, not all patients can benefit from the clinical managements aiming at immuno-suppression or melanocyte regeneration [13–15]. Especially for patients of segmental subtype at late stage with lesional leukotrichia or lesions on glabrous skin,photo-therapy is not an appropriate strategy. When the disease is stable, surgical treatment is a valuable alternative [10].

Surgical techniques usually aim to provide melanocytic cells to previously depigmented areas [16] by subdividing them into tissue grafts and cellular grafts (cultured melanocytes and non-cultured epidermal cellular grafts) [12, 17, 18]. Among them, autologous non-cultured melanocyte-keratinocyte transplantation procedure (MKTP) is one of the simplest cellular grafting techniques, and it is currently the most popular among dermatologists [19]. It offers 50%–100% re-pigmentation rates with donor-to-recipient ratio between 1:3 to 1:10, showing acceptable color matching in most of the treated cases [20–24]. Since it was first developed by Gauthier and Surleve-Bazeille in 1992 [25], a number of studies have been done on the effectiveness of MKTP in treating stable vitiligo [10, 12, 19]. However, studies with large database and long-term follow up, especially the transplantation result after six years or longer, were rare.

It is known that the response to MKTP in general is affected by several factors. As with other surgical techniques, proper patient selection and exquisite technical skills are of equal importance in MKTP. Vitiligo stability is considered as a vital parameter before considering any melanocyte transplantation technique. We found that different durations of disease stability are employed as inclusion criterion in studies applying MKTP in vitiligo treatment. Some authors considered 6 months to be sufficient [26–28], while others required 1 year of disease stability [23, 29]. Olsson even suggested patients who have not had stable, non-progressive vitiligo for at least 2 years should not be chosen for transplantation [26].

In this study, we applied MKTP as treatment of 2283 cases of vitiligo and followed up for a period of 1-9 years. We retrospectively evaluated the long-term re-pigmentation after MKTP with the aim to identify the associated factors for successful outcome.

## RESULTS

### Patient characteristics

The analysis is comprised of 2283 patients who had long-term follow-up data (12-108 months;mean:43 months; median:36 months). Table 1 illustrates the demographic and vitiligo characteristics at baseline. 2171 patients (94. 4%) were followed up for at least 2 years. There were 2283 patients with different types of vitiligo (973 males, 1310 females, mean age 24. 1 years, range 4-63 years, 1716 patients aged <30 years). The duration of vitiligo varied from 1 to 40 years, with a mean duration of 6.0 years. 606 patients (26. 5%) had segmental involvement; 1091 patients (47. 7%) had non-segmental vitiligo, and 589 patients (25. 8%) had undefined vitiligo (focal). The maximum area operated in one individual patient was 200cm^2^ and the minimum was 2 cm^2^. 79 patients (3. 4%) had a family history of vitiligo, and 123 patients (5. 4%) had a Koebner phenomenon. The face and neck were the two most transplanted sites, followed by the back of hand.

**Table 1:** Patients’ characteristics.

**Gender**	
male	973(42.6%)
female	1310(57.4%)
Age (years), mean ±SD (median; range)	24.1±11.1 (22;4-63)
Vitiligo Pattern, n (%)	
Segmental	606(26.5%)
Non-segmental	1088(47.7%)
Undefined(focal)	589(25.8%)
Duration of disease (years), mean ±SD (median; range)	6.0±5.8 (4;1-40)
Stability time (months), mean ±SD (median; range)	23.5±39.3 (12;6-480)
Treated area surface (cm^2^), mean ±SD (median)	36.3±27.3 (30;2-200)
Koebner phenomenon, n (%)	123(5%)
Family history of vitiligo, n (%)	79(3%)
Follow-up period, n (%)	
1 year	127(5.6%)
2 year	624(27.3%)
3 year	560(24.5%)
4 year	471(20.6%)
5 year	293(12.8%)
6 year	115(5.0%)
7 year	43(1.9%)
8 year	38(1.7%)
9 year	12(0.5%)

### Re-pigmentation results

**Figure 1:**
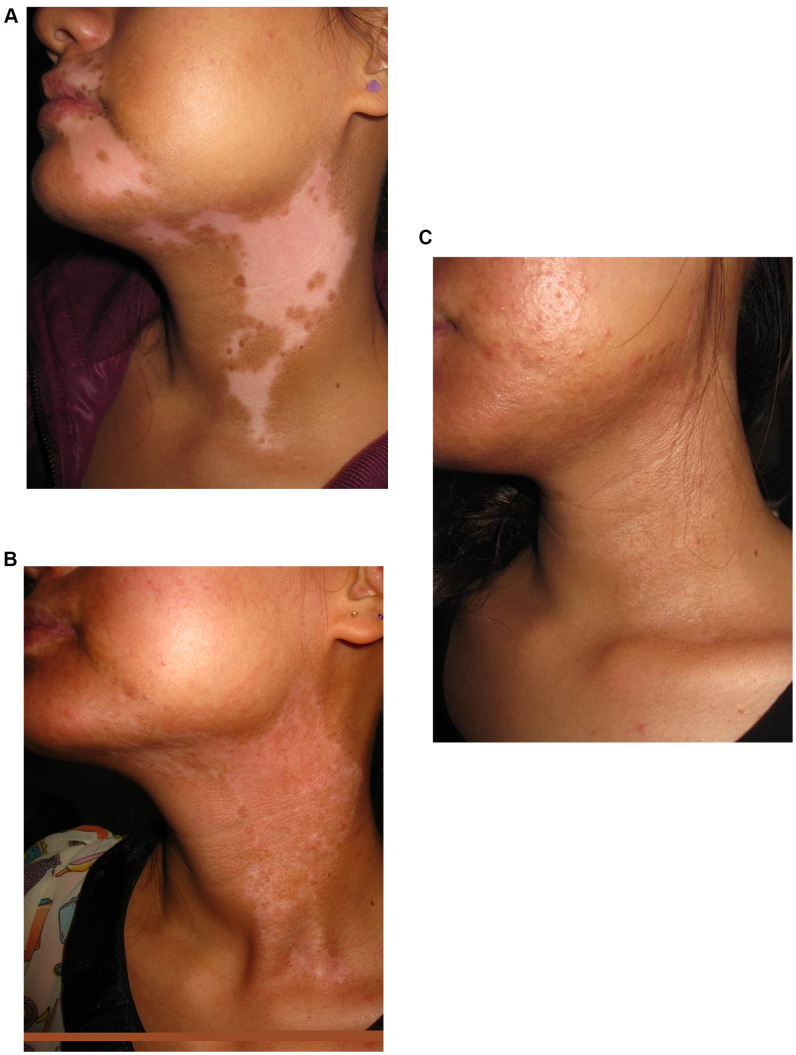
**A 20-year old woman with segmental vitiligo on the face and neck.** (**A**) Before surgery. A 20-year old woman with segmental vitiligo on the face and neck. (**B**) Six months after autologous non-cultured melanocyte-keratinocyte transplantation procedure (MKTP). A 20-year old woman with segmental vitiligo on the face and neck. (**C**) Twelve months after MKTP with a repigmentation of 100%.

**Figure 2:**
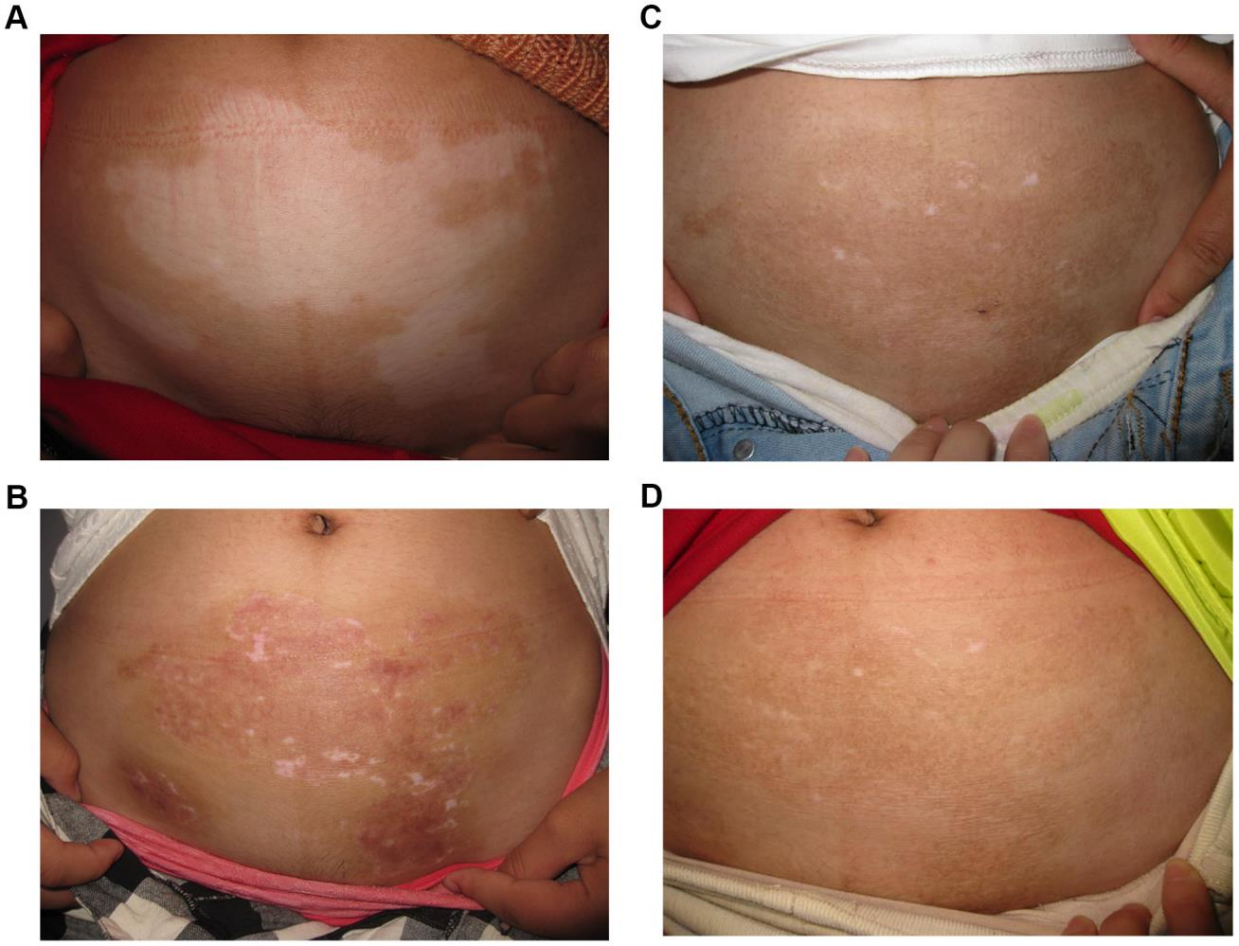
**A 28-year old woman with non-segmental vitiligo on the abdomen.** (**A**) Before surgery. A 28-year old woman with non-segmental vitiligo on the abdomen. (**B**) Three months after autologous non-cultured melanocyte-keratinocyte transplantation procedure(MKTP). A 28-year old woman with non-segmental vitiligo on the abdomen. (**C**) Six months after MKTP with a regimentation of 98%. A 28-year old woman with non-segmental vitiligo on the abdomen. (**D**) Twelve months after MKTP with a repigmentation of 98%.

**Figure 3:**
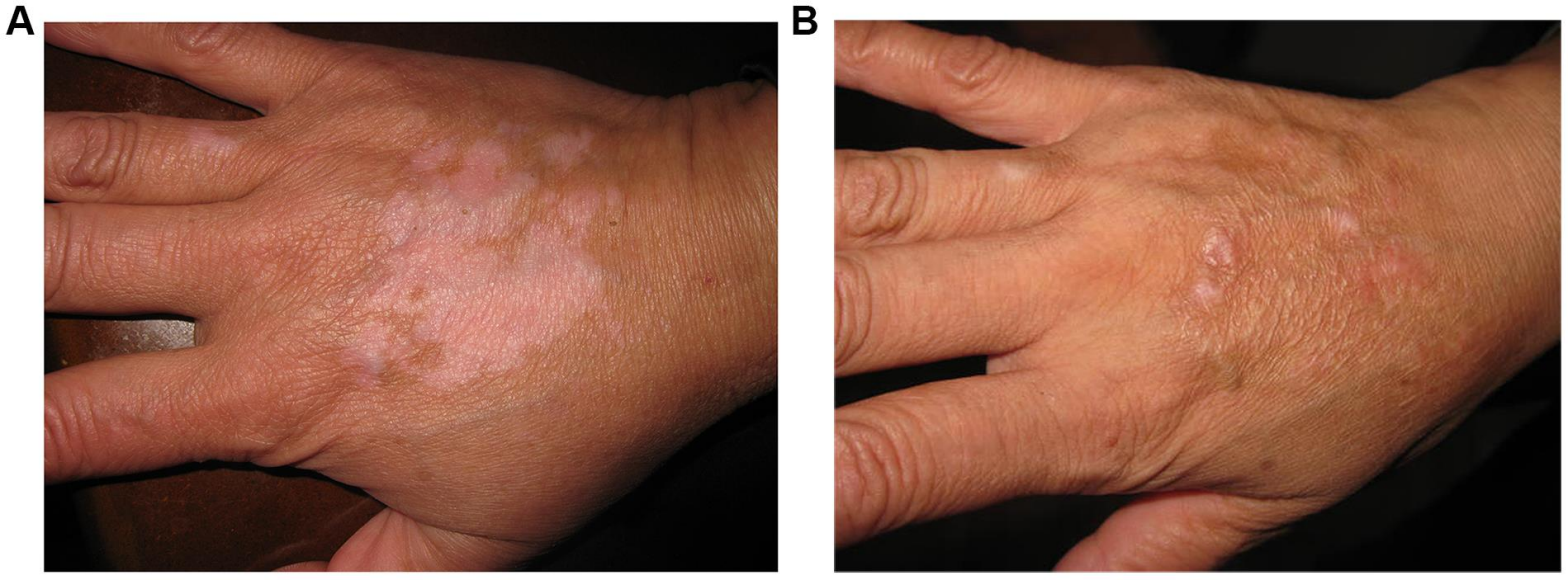
**A 29-year old man with non-segmental vitiligo on the back of hand.** (**A**) Before surgery. A 29-year old man with non-segmental vitiligo on the back of hand. (**B**) Two years after autologous non-cultured melanocyte-keratinocyte transplantation procedure.

Lesion with excellent re-pigmentation was considered to be successful (Figures 1–3). Excellent re-pigmentation was achieved in 400/606 (66%), 315/589 (53. 5%), and 506/1088 (46. 5%) in patients with segmental vitiligo (SV), undefined vitiligo (focal) and non-segmental vitiligo (NSV), respectively. Obviously, the response of SV was better than the others, and there was a significant difference among them (χ^2^=72. 698, P<0. 001).

Evaluation of re-pigmentation outcomes in different age groups showed a statistical difference between the younger group and the elder. 788/1341 (58. 8%) patients who were younger than 24 years achieved better re-pigmentation when to the group of >24 years with the success rate of 47. 5% (χ^2^=40. 888, *P*<0. 001). However, multivariable analysis showed that age was not a statistically significant covariate associated with treatment success.

Analysis of anatomic location is shown in Table 2. More than half of the patients with the lesion on the face and neck, trunk and limbs attained excellent re-pigmentation, though no significant difference was found among these sites. The region of perineum and scrotum responded well to the transplantation, and 18/24 patients (75%) achieved excellent re-pigmentation. It is more difficult to achieve satisfying results in elbow, knee, acral, eyelid, and mucosa, as the excellent re-pigmentation rates were 23.1%, 32.5%, 33.3% and 40%, respectively. Yet there was a significant difference in re-pigmentation between the historically reported difficult-to-treat areas and non-difficult areas (χ^2^=74.872, *P*<0.001).

**Table 2:** Long-term repigmentation results in patients with different associated factors.

	**Number of lesion**	**Repigmentation result**				***P* value**
		**excellent**	**good**	**fair**	**poor**	
Vitiligo type						
Segmental vitiligo	606	400	135	41	30	
Non-segmental vitiligo	1088	506	257	132	193	*P*<0.001
Undefined vitiligo(focal)	589	315	183	49	42	
Family history						
No	2204	1196	552	214	242	*P*<0.001
Yes	79	25	23	8	23	
NB-UVB before surgery						
No	1599	784	449	180	186	*P*<0.001
Yes	684	437	126	42	79	
Anatomically based lesions						
scalp	32	19	5	1	7	
face and neck	918	529	230	76	83	
trunk	693	382	168	69	74	
limb	304	169	79	26	30	
acral	255	83	72	41	59	*P*<0.001
eyelid	21	7	8	1	5	
mucosa(lip and areola)	10	4	2	3	1	
perineum and scrotum	24	18	4	0	2	
elbow and knee	13	3	4	3	3	
Multiple sites	13	7	3	2	1	
Stability time						
6months	889	410	213	113	153	
7-12months	608	332	153	54	69	
13-18months	93	60	24	5	4	
19-24months	241	145	56	21	19	*P*<0.001
25-30months	56	36	16	3	1	
31-36months	96	64	22	4	6	
>37months	300	174	91	22	13	

With increasing follow-up intervals, a gradual increase in the extent of re-pigmentation was noted. More than half of patients could attain additional re-pigmentation even 9 months after surgery. Only a few patients were noted partially to completely reduced pigmentation. Unexpectedly, 30 patients developed new lesions, while the operated sites were still retaining pigmentation. 208 patients were followed up for more than 72 months, and 206 of them retained re-pigmentation. Even with the longest follow-up period of 108 months, the acquired pigmentation was still maintained in 11/12 patients.

In this study, there were nearly one third of the patients who had received NB-UVB therapy before the MKTP. 437/684 (63.9%) patients with pre-MKTP phototherapy achieved excellent re-pigmentation, compared to 784/1599 (49.0%) patients without phototherapy. There was a statistically difference between the pre-MKTP phototherapy group and group without (χ^2^=50. 422, *P*<0.001), and the results also revealed that NB-UVB can accelerate the re-pigmentation.

Stability time was an important factor influencing the treatment results. A significant positive correlation between duration of stability and percentage re-pigmentation of the lesions was found (χ^2^=42. 053, *P*<0. 001). In multivariable analysis, duration of stability longer than 12 months (compared with 6 months; OR 1. 913, *P*=0. 007), together with vitiligo type, family history, site of lesion and combination of pre-MKTP phototherapy, were identified to be an independent factor associated with successful outcome.

The size of treated area seemed to have no influence on the transplantation result. The patients with a positive family history responded poorer than the others (χ^2^=29. 417, P<0. 001). Koebner phenomenon (84. 6%) happened mostly in patients with a duration of stability less than 12 months, and those patients showed a poorer response (χ^2^=107. 397, P<0. 001).

## DISCUSSION

The non-cultured MKTP was invented by Mulekar based on the procedure of Olsson and Juhlin in 2004 [22, 23]. Using this technique, Mulekar attained over 95% re-pigmentation in 84%, 73%, and 56% of 50 segmental, 17 focal, and 142 generalized vitiligo patients, respectively [22, 23, 30]. In 2015, Bao et al. observed 53% of excellent re-pigmentation and 28% of good re-pigmentation in patients with segmental and non-segmental vitiligo with non-cultured MKTP treatment [30]. Our study of MKTP using roofs of suction blisters for suspension preparation and ultra-pulse CO_2_ laser for recipient-site dermabrasion is the largest-scale up to now. It was encouraging to see that excellent re-pigmentation was achieved in more than half of the lesions and the pigment retained at the end of the respective follow-up period. Statistical analyses showed that vitiligo type, family history, site of lesion, pre-grafting NB-UVB therapy, and duration of disease stability were all the influential factors for a successful outcome.

The technique of obtaining the donor skin in our study differed from most of the previous studies. Most researchers used split-thickness donor skin that were taken by a shaving blade, while we used the roof of suction blisters. Although suction blister formation is time-consuming, the incubation period of skin sample in trypsin is shortened, and it rarely leads to development of textural change or scar in the donor site.

Consistent with previous studies [20, 22, 31], SV achieved better outcomes when compared with NSV. Almost all vitiligo patients with MKTP reported recalcitrance to standard interventions of topical corticosteroids/calcineurin inhibitors and phototherapy. The differences in transplantation results could be explained by an etiological difference [26].

The influence of age at surgery on the treatment result remains unclear. Chen et al. reported that adult patients younger than 40 years had better outcomes [32]. However, after adjustment for different categories of vitiligo, the influence of age was not significant [32]. Olsson and colleagues reported that younger patients seem to respond better to the treatment, though no statistical analyses were carried out to verify the significance of this difference [33]. In this study, we found age was not a statistically significant covariate associated with treatment success.

The family history of vitiligo can significantly affect the therapeutic outcome of MKTP. Our data showed that 79 patients with a positive family history responded worse than others with no family history. The individuals who had first-degree relatives with vitiligo have higher risk for developing the disease: nearly 6% compared to 1% or less in the general population. The genome-wide association studies (GWAS) confirmed that nearly 50 genetic loci are closely associated with the initiation of vitiligo [2, 34]. Therefore, we hypothesized that there are certain genes that could affect the re-pigmentation after MKTP.

When the relation between transplanted site and the outcome was analyzed, there was no statistical difference among the site of face and neck, trunk and limbs. However we found significant difference in re-pigmentation between historically reported difficult-to-treat areas and non-difficult areas. In a recent study, 35% of acral lesions (excluding those on fingertips and toes) demonstrated excellent re-pigmentation [35]. Similarly, lesions over the joints, including elbows, knees and ankles, tend to respond less favorably with less than 30% of excellent re-pigmentation [36]. Our results were consistent with the previous reports.

It is widely known that vitiligo on the lips and the tip of fingers and toes (the so-called lip-tip vitiligo) is less responsive to any treatment [37]. Huggins et al. showed that over 70% of patients with lip-tip vitiligo attained poor re-pigmentation [18]. Inadequate depth of dermabrasion due to heavily cornified skin as well as the high mobility at these sites may explain the poor response [19].

The result that re-pigmentation was improved by pre-MKTP phototherapy was not surprising. We had previously reported that combination with NB-UVB therapy increased the effectiveness of cultured autologous melanocytes transplantation as well [38]. Over the years, several studies have utilized post-transplantation phototherapy to enhance re-pigmentation [18, 36], though no study was carried out to evaluate the role of pre-MKTP phototherapy. Here we confirmed its enhanced role in the re-pigmentation outcome. We thought it might be explained by the effect of NB-UVB in depleting skin-infiltrating T cells through the induction of apoptosis [39], so it may be better for vitiligo patients to undergo NB-UVB therapy before MKTP. Mutalik et al. suggested that postoperative addition of CsA may contribute to enhanced and uniform pigmentation, achieving a perfect blend with surrounding skin and acceptable aesthetic results [40]. In addition, postoperative care is also vital for the success of the treatment [41]. For example, collagen dressing can promote the adherence and survival of the transplanted cells [41].

The large differences in the transplantation effect between patients who had shorter stability time (6-12 months) and those with longer stability time (>12 months) strongly indicate the importance of stability before conducting a transplantation. This points to the need for careful selection of vitiligo patients. Rao et al., found a significantly higher lesioned CD8^+^ T-cell counting vitiligo patients with shorter duration of stability (3-12months) in comparison with those with longer duration of stability (>12months) [42]. Both Rao et al and Zhou et al observed patients with high numbers of perilesional CD8^+^T cells showed poor rate of re-pigmentation, concluding that the activity of cytotoxic CD8^+^cells in vitiligo lesions may be responsible for inferior post-transplant outcome [42, 43].

Although consensus regarding the ideal period of stability is under-studied, we recommend the duration of clinical stability being longer than 1 year. In clinical practice, dermatologists often rely on the history and certain clinical features to judge disease progression, which is considered grossly inaccurate. In recent years, the non-invasive real-time *in vivo* reflectance confocal microscopy (RCM) imaging has been reported an efficient and reliable tool to improve the accuracy of staging vitiliginous lesion [44]. With a better selection of cases for MKTP, a superior re-pigmentation result is promising.

However, this is a retrospective study as we did not pay enough attention to the color matching and detailed adverse events in the follow up visit. There was no record of the total areas of vitiligo lesion. And the influence of Fitzpatrick skin type of the patients on the outcome was neglected. Further, we did not analyze the postoperative relapse rates, which is related to the prognosis of the patient. Gan et al. reported that a relapse in 11% of all 177 patients, and the relapse was defined by occurrence of de-pigmentation of previously grafted area, other sites, or both [36]. Altalhab et al., suggested that a larger BSA involvement and fingertip involvement were associated with higher relapse rates [45]. Therefore, these clinical signs should be carefully observed before performing any surgical procedure as it is of poor prognosis.

MKTP holds therapeutic promise and has proven to be a safe and effective treatment for patients. Further improvement of the transplantation technique may lead to better outcomes even in difficult-to-treat areas.

## MATERIALS AND METHODS

This retrospective review was approved by the Medical Ethics Committee of Third People’s Hospital of Hangzhou. Electronic medical records of all patients who underwent MKTP at the Department of Dermatology, the Third People’s Hospital of Hangzhou between June 2009 and June 2018 were retrieved. Patients were followed up until June 2019. The clinical information of all patients was available for at least 12 months after the procedure. Inclusion criteria for MKTP were stable vitiligo (static lesions for at least 6 months and absent Koebner phenomenon) failing to respond to conventional topical and systemic therapies. Patients with pregnancy, history of keloid formation, and bleeding diathesis were excluded. No wash out period was required for patients receiving topical or systemic medication.

Clinical and demographic data, characteristics, and type of vitiligo were obtained before the surgical procedure. Routine clinical photographs were obtained for all patients before MKTP and at each follow-up visit. The percentage of re-pigmentation was evaluated by physicians at month 3,6,9,12 and yearly after. Re-pigmentation of more than 90% was graded as excellent if the, good if between 50 and 89%, fair if the between 20 and 49%, and poor if the re-pigmentation was less than 20%.

### Melanocyte– keratinocyte transplantation procedure

The entire procedure included suction blisters in the donor area, separation of cells, preparation of recipient area, and transplantation of cell suspension on recipient area.

### Preparation of cell suspension

Donor skin was obtained from a normal pigmented area of the abdomen or the lateral aspect of the gluteal region. Blisters of 0.80 cm^2^ each were produced using suction (skin separating instruments; Model BFY-IIA; Satellite Medical Equipment Made (Shaoxing) Co. Ltd., Shaoxing, China) with a vacuum of 300 mmHg for 1 to 2 hours, depending on the speed of blister formation. The size of the donor skin was taken approximately as one-third to one-tenth of the recipient area. The roofs of the blisters were excised and sent for cell separation. Specimens were washed with calcium-free Hanks’ solution (D-Hanks’ solution) and incubated in 0. 25% trypsin solution for 10 minutes, followed by incubation with 0. 02% EDTA solution for 10 minutes at 37° C. Cells were separated from the epidermal sheet under a stereoscopic microscope. The cell suspension was centrifuged, and resuspended with F12 medium.

### Transplantation procedure

Lidocaine cream was applied topically to the recipient sites 2 hours before transplantation. In more-sensitive patients, injection of 1% lidocaine was used for local anesthesia. The recipient areas were then cleaned with 75% alcohol and treated with an ultra-pulse CO_2_ laser (power 30-50 Hz/s, energy 225 mJ/s) to remove the epidermis by superficial abrasion for 1-2 passes. Normal saline-soaked gauze was used to remove the whitish epidermis after laser passes, and the dermis was avoided to prevent scar formation. The melanocyte suspension was spread evenly to the denuded recipient area. Then, the recipient area was covered with Vaseline gauze followed by gauze soaked with F12 medium and secured with gauze pieces and surgical tape. After the procedure, patients were instructed to lie still in the same position for at least 1 hour to ensure cell fixation before patients were permitted to go home and instructed not to scrub the areas and avoid all vigorous physical activities. No medication was prescribed post-procedure, and dressings were removed after 7-10 days.

### Statistical analysis

The chi-square distribution test was used to assess the significance of differences between various groups. Multivariate logistic regression analysis was used to determine the importance of various variables on the transplant results. *P*<0. 05 was considered to be statistically significant. All analyses were performed using SPSS17.
